# Evidence for potassium transport activity of Arabidopsis KEA1-KEA6

**DOI:** 10.1038/s41598-019-46463-7

**Published:** 2019-07-11

**Authors:** Masaru Tsujii, Kota Kera, Shin Hamamoto, Takashi Kuromori, Toshiharu Shikanai, Nobuyuki Uozumi

**Affiliations:** 1 Department of Biomolecular Engineering, Graduate School of Engineering, Tohoku University, Aobayama 6-6-07, Sendai, 980-8579 Japan; 2 RIKEN Center for Sustainable Resource Science, Wako, Saitama 351-0198 Japan; 3 Department of Botany, Graduate School of Science, Kyoto University, Kyoto, 606-8502 Japan

**Keywords:** Potassium channels, Plant molecular biology

## Abstract

*Arabidopsis thaliana* contains the putative K^+^ efflux transporters KEA1-KEA6, similar to KefB and KefC of *Escherichia coli*. KEA1-KEA3 are involved in the regulation of photosynthetic electron transport and chloroplast development. KEA4-KEA6 mediate pH regulation of the endomembrane network during salinity stress. However, the ion transport activities of KEA1-KEA6 have not been directly characterized. In this study, we used an *E*. *coli* expression system to examine KEA activity. KEA1-KEA3 and KEA5 showed bi-directional K^+^ transport activity, whereas KEA4 and KEA6 functioned as a K^+^ uptake system. The thylakoid membrane-localized Na^+^/H^+^ antiporter NhaS3 from the model cyanobacterium *Synechocystis* is the closest homolog of KEA3. Changing the putative Na^+^/H^+^ selective site of KEA3 (Gln-Asp) to that of NhaS3 (Asp-Asp) did not alter the ion selectivity without loss of K^+^ transport activity. The first residue in the conserved motif was not a determinant for K^+^ or Na^+^ selectivity. Deletion of the possible nucleotide-binding KTN domain from KEA3 lowered K^+^ transport activity, indicating that the KTN domain was important for this function. The KEA3-G422R mutation discovered in the Arabidopsis *dpgr* mutant increased K^+^ transport activity, consistent with the mutant phenotype. These results indicate that Arabidopsis KEA1-KEA6 act as K^+^ transport systems, and support the interpretation that KEA3 promotes dissipation of ΔpH in the thylakoid membrane.

## Introduction

Tight control of intracellular pH and ion concentrations through the activity of membrane transport systems is crucial for maintaining intracellular ion homeostasis during changes in the environment and for allowing the formation of an electrochemical potential across the membrane. In chloroplasts and cyanobacteria, the flux of protons and other ions across the thylakoid membrane is highly correlated with light energy conversion in the photosynthetic electron transport system. Light reactions generate proton (H^+^) motive force (pmf), which is the sum of a pH gradient component (ΔpH) and an electric potential component (∆Ψ) across the thylakoid membrane. Since the potassium ion (K^+^) is the major essential cation in most living cells, including plant cells and cyanobacteria, K^+^ concentration and K^+^ flux across the membrane conducted by K^+^ transport systems are a main contributor to the electric potential component ∆Ψ^1–3^ and to the intracellular osmotic homeostasis in response to external osmotic changes.

Several K^+^ transporters are present in chloroplasts and in cyanobacteria. In *Synechocystis* sp. PCC 6803, two types of K^+^ uptake transporters, KtrB and KdpA^4,5^ and K^+^ channels such as SynK^6,7^ and SynCaK^8^ have been reported. Using antibodies, SynK has been shown to be present both in fractions of thylakoid membrane and plasma membrane. In chloroplasts of *Arabidopsis thaliana*, the two-pore K^+^ channel TPK3 was detected in the thylakoid membrane fraction as well^6^. Upon further examination TPK3 was found to reside in the thylakoid stromal lamellae^9^. Recent studies also revealed that putative K^+^/H^+^ antiporters, KEA1-KEA3, are expressed in Arabidopsis chloroplasts^10^.

Arabidopsis has six genes encoding KEAs^11,12^. KEAs belong to the monovalent cation/proton antiporter (CPA) super family. The CPA family consists of two subfamilies, CPA1 and CPA2; the Na^+^-H^+^ exchangers (NHX) belong to the CPA1 family, and the K^+^ efflux antiporters (KEA) and cation-H^+^ exchangers (CHX) belong to the CPA2 family. The Arabidopsis NHXs have been studied in great detail; NHX1-NHX6 reside in the endosome membrane, and NHX7(SOS1)-NHX8 function in the plasma membrane^13^. NHXs play a critical role in salt tolerance and pH homeostasis. Characterization of KEA1-KEA6 has recently been reported by several groups but detailed information on the function of these transporters remains elusive^10,14–20^.

The KEAs can be divided into two clades, KEA1-KEA3 (KEAI) and KEA4-KEA6 (KEAII), based on an alignment of their protein sequences^12^. KEA1 and KEA2 belong to KEA-Ia, and KEA3 belongs to KEA-Ib^12^. KEA-Ia is characterized by a longer N-terminal sequence. KEA1-KEA3 also possess a putative chloroplast transit peptide in their N-terminal regions. Biochemical approaches confirmed that KEA1 and KEA2 are localized in the inner envelope of the chloroplast, and that KEA3 is present in the thylakoid membrane^10,14^. A *kea1 kea2* double as well as a *kea1 kea2 kea3* triple mutant have swollen and shortened chloroplasts, and are hypersensitive to salt stress^10,19^. KEA3 accelerates the non-photochemical quenching (NPQ) relaxation after transition from high to low light^14,15,17^. A recent study reported that KEA4-KEA6 are localized in the endomembrane network composing the trans-Golgi network and the pre-vacuolar compartment/multivesicular bodies^20^. A *kea4 kea5 kea6* triple mutant accumulates 1.4-fold more Na^+^ compared with the wild type in medium containing 100 mM NaCl. KEA4-KEA6 may promote plant growth and enhance salinity tolerance by contributing to pH maintenance of the endomembrane network.

Prior to recent genetic studies of Arabidopsis KEAs, KEA homologs from the model cyanobacterium *Synechocystis* PCC 6803, NhaS1-NhaS6, have been investigated. According to the endosymbiont theory, chloroplasts evolved from cyanobacteria that were taken up into early eukaryotic cells. Therefore, information collected on the function of the well-characterized *Synechocystis* NhaS1-NhaS6 transporters may provide an insight into the function of KEA1-KEA6 in Arabidopsis. NhaS1, also known as SynNhaP, functions as a Na^+^/H^+^ antiporter^21^. A single knockout mutants of *nhaS2* and *nhaS4* grows more slowly in low-Na^+^ concentrations, indicating that NhaS2 and NhaS4 function in Na^+^ uptake^22^. NhaS3, which is the closest homolog to KEA3 in *Synechocystis* PCC6803 (identity: 25%, similarity: 65%), is an essential Na^+^/H^+^ antiporter in thylakoid membrane because a null mutant of the *nhaS3* gene could not be generated^23^. NhaS3 confers tolerance to external high osmolarity, and is required for proper regulation of cytosolic and luminal pH^23^. NhaS3 likely controls pmf by alleviating the excess reduction status generated by the light reaction. Based on this evidence, *Synechocystis* NhaS3 and chloroplast KEA3 are likely to share a similar physiological role in the thylakoid membrane.

The K^+^ efflux system consisting of KefB and KefC in *E*. *coli*, is thought to be the functional equivalent of KEA1-KEA6^12^. The K^+^ transport nucleotide binding (KTN) domain located in the C-terminal region of KefB/KefC is involved in the regulation of K^+^ transport activity via glutathione and NAD(P)H binding^24,25^. The KTN domain is present in KEA1-KEA3, but absent from KEA4-KEA6. Therefore, it is plausible that regulation of KEA4-KEA6-mediated transport activity may be different from that of KefB/KefC^20^.

Although the physiological role of the KEAs has been studied, functional data of KEA1-KEA6 remains limited. Heterologous expression in a *Saccharomyces cerevisiae* potassium transport mutant and transport activity measurements with reconstituted microsomes indicate that KEA2 has K^+^/H^+^ activity^18^. KEA4-KEA6 are able to complement the *E*. *coli nhaA nhaB* mutant and a *kefB kefC* mutant^20^. Therefore, KEA2 and KEA4-KEA6 are considered to function as K^+^/H^+^ antiporters. Despite these results, the transport properties of KEA1-KEA6 have not yet been studied directly. In this study, we characterized KEA transport activity by heterologous expression in *E*. *coli*^26^. We took advantage of an *E*. *coli* expression system to determine K^+^ transport activity of a plant transport system because K^+^ channels and transporters located in the endosome membrane in plant cells locate to the cell membrane when expressed in *E*. *coli*^26^. This method has been successfully used to determine the function of the ancestral cyanobacterial Na^+^/H^+^ antiporter NhaS3^23^. The assays performed in this study demonstrate the ion transport characteristics of KEA1-KEA6.

## Results

### Identification of KEA1-KEA6-mediated K^+^ transport activity

**Figure 1:**
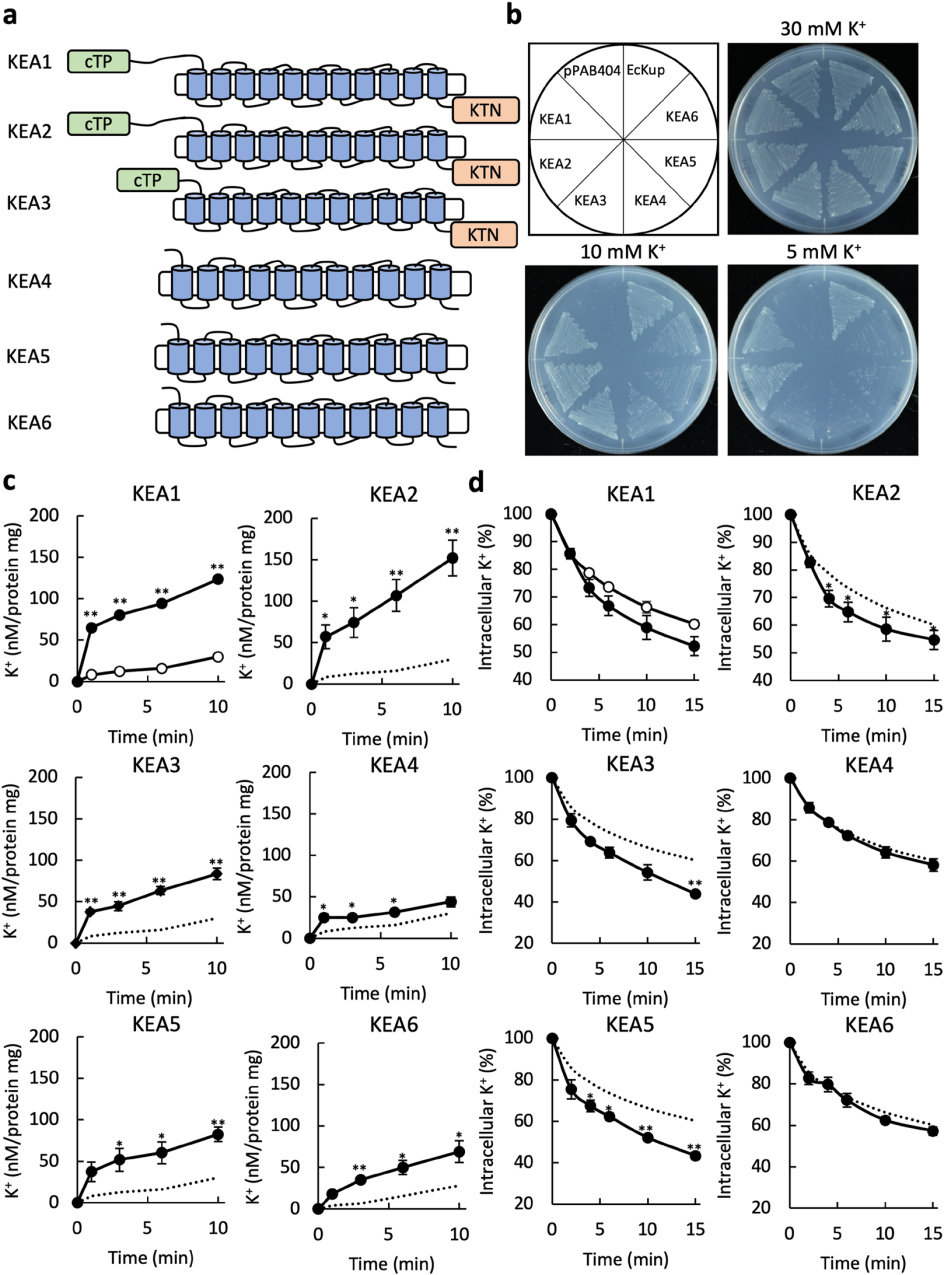
K^+^ transport activity of KEA1-KEA6. (**a**) Predicted membrane topology of KEA1-KEA6. KEA1-KEA3, are localized in chloroplasts of *Arabidopsis thaliana*^10^ and possess a chloroplast transit peptide (cTP) at their N-terminus and a K^+^ transport nucleotide binding (KTN) domain at their C-terminus. KEA4-KEA6 may not be localized in chloroplasts. Prediction programs used were ChloroP 1.1 (http://www.cbs.dtu.dk/services/ChloroP/) for chloroplast transit peptide and SOSUI (http://harrier.nagahama-i-bio.ac.jp/sosui/) for transmembrane domains. (**b**) Growth complementation assay of an *E*. *coli* K^+^ uptake deficient mutant (LB2003) expressing KEA1-KEA6. Transformants were grown on synthetic medium supplemented with 5, 10 or 30 mM KCl at 30 °C for 24 h. Cells transformed with *E*. *coli* Kup (EcKup) or the empty vector were included as positive and negative control, respectively^51^. (**c**) K^+^ uptake activity into LB2003 containing KEA1-KEA6 (closed symbols) or the empty vector pPAB404 as negative control (open symbols in the KEA1 graph or broken lines in the rest). The data for the control shown in each graph is the same. For the assay, KCl was added to a final concentration of 10 mM to the cells suspended in 200 mM HEPES-NaOH (pH 8.0). Error bars indicate SD (**p < 0.01, 0.01 < *p < 0.05, n = 3). (**d**) K^+^ efflux rate from the same transformants as in (**c**). Cells (16 μL) were added to 200 μL of a solution containing 0.4 M NaCl and 20 mM HEPES-NaOH (pH 8.0) at t = 0. Error bars indicate SD (**p < 0.01, 0.01 < *p < 0.05, n = 3). The symbols are the same as in (**c**).

We isolated the cDNAs of *KEA1-KEA6* and introduced them into the K^+^-uptake deficient *E*. *coli* strain LB2003^27^. KEA1, KEA2 and KEA3 contain an extended N-terminal region in addition to the chloroplast transit peptide, located in the stroma (Fig. 1a and Supplementary Fig. S1). Earlier attempts at functional expression of KEA2 in *E*. *coli* failed, presumably due to cytotoxicity of the full-length KEA2 protein^18^. To avoid this problem, we used versions of KEA1-KEA3 with truncated N-terminal regions for this study. KEA3 has three splice variants (described below). Unless otherwise noted, in our study KEA3 refers to KEA3.2, the splice variant with the longest C-terminal region. A previous study reported a failure of expression of the genes encoding full-length KEA4-KEA6 in *E*. *coli*^20^. For our study, we carefully tested conditions for expressing full-length KEA4-KEA6 in the *E*. *coli* mutant. *E*. *coli* LB2003 expressing KEA1-KEA6 was able to grow as well as the positive control expressing EcKup^28^ in media containing 10 mM KCl (Fig. 1b). KEA1 and KEA2 rescued the growth of the *E*. *coli* LB2003 mutant in the presence of 5 mM KCl. Cells expressing KEA1-KEA6 had higher K^+^ uptake transport activity than the same strain transformed with the empty vector (Fig. 1c). KEA1 and KEA2 showed the highest K^+^ uptake activity. For the determination of K^+^ efflux activity, KEA1-KEA6 were expressed in *E*. *coli* strain TO114, which possesses low K^+^ export activity due to its lack of Na^+^(K^+^)/H^+^ antiporters, NhaA, NhaB and ChaA^29^. The K^+^ efflux rate from cells expressing KEA1, KEA2, KEA3 or KEA5 was higher than that of cells containing the empty vector. No significant K^+^ efflux was observed with KEA4 and KEA6 in this assay (Fig. 1d).

### Na^+^ uptake activity of KEA1, KEA2 and KEA3

**Figure 2:**
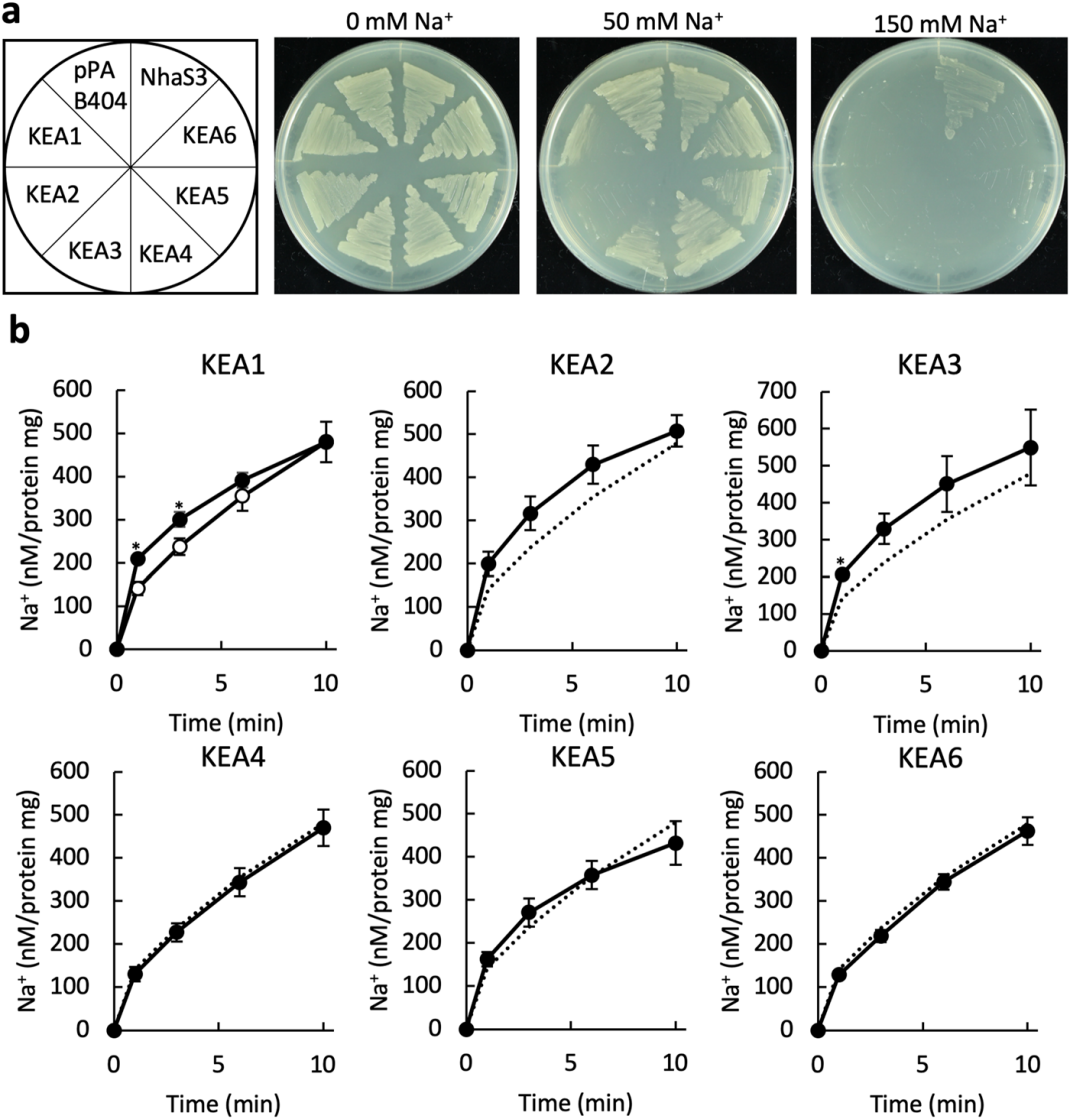
Na^+^ transport activity of KEA1-KEA6. (**a**) Complementation assay of *E*. *coli* TO114 containing plasmids encoding KEA1-KEA6 or the empty vector pPAB404. Transformants were grown on medium supplemented with 0, 50 or 150 mM NaCl at 30 °C for 24 h. NhaS3, a *Synechocystis* sp. PCC 6803 Na^+^/H^+^ antiporter, was used as a positive control^23^. (**b**) Na^+^ uptake by *E*. *coli* TO114 containing KEA1-KEA6 (closed symbols) or pPAB404 (open symbols) as a negative control. The broken lines represent the data obtained with pPAB404 shown in the graph on the left. NaCl was added to a final concertation of 10 mM to the cells suspended in 200 mM HEPES-TEA (pH 8.0). Error bars indicate SD (**p < 0.01, 0.01 < *p < 0.05, n = 3).

We assessed Na^+^ transport activity using *E*. *coli* strain TO114, which is sensitive to high salinity due to loss of function of three Na^+^(K^+^)/H^+^ antiporters. KEA1-KEA6 failed to complement the high salinity sensitivity of the TO114 strain in medium containing 150 mM NaCl. Under the same conditions, the cyanobacterial Na^+^/H^+^ antiporter, NhaS3, a homolog of the KEAs was able to rescue the lack of Na^+^ extrusion of the TO114 strain^23^ (Fig. 2a). Cells expressing KEA1, KEA2 and KEA3 showed higher sensitivity to mild salinity stress (50 mM NaCl), suggesting that these proteins functioned as Na^+^ uptake transporters. To confirm this, we measured the Na^+^ accumulation rate in the cells (Fig. 2b). The Na^+^ content of cells expressing KEA1-KEA3 was slightly higher but not significant compared with that of the control cells containing the empty vector. Cells expressing KEA4-KEA6 did not show Na^+^ accumulation or Na^+^ extrusion. The data showed that the overall Na^+^ translocation rate mediated by KEA1-KEA6 was zero or very low, unlike their K^+^ transport activity.

### Measurement of cation-proton antiporter activities of KEA1-KEA6

**Figure 3:**
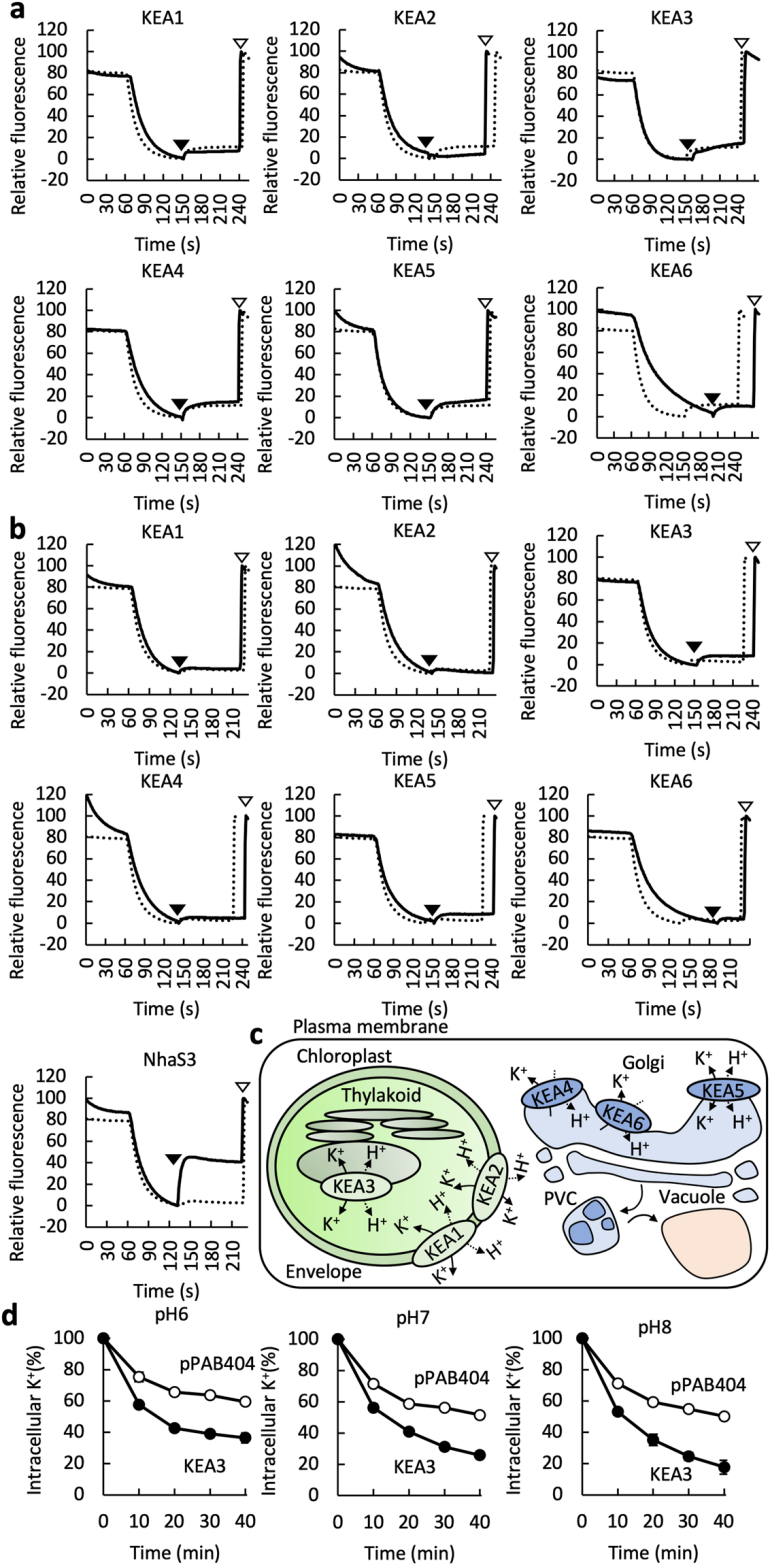
K^+^/H^+^ and Na^+^/H^+^ antiporter activity of KEA1-KEA6. (**a**) Representative profile of K^+^/H^+^ antiporter activity of KEA1-KEA6 in *E*. *coli* membrane vesicles. The percentage of fluorescence dequenching elicited by the addition of 8 mM KCl (closed triangle) was plotted relative to that of NH_4_Cl-induced dequenching (open triangle). Solid lines represent the results of KEA1-KEA6 and dashed lines indicate the data obtained with the negative control (empty vector) under the same conditions. (**b**) Representative profile of Na^+^/H^+^ antiporter activity of KEA1-KEA6 and NhaS3. The assay was performed as for (**a**) except that NaCl was added instead of KCl. (**c**) Model summarizing the cellular localization and function of KEA1-KEA6. (**d**) pH dependency of KEA3-mediated K^+^ efflux by *E*. *coli* TO114 cells. The measurement was performed in the same way as in Fig. 1(d) but with different buffers; 10 mM MES-NaOH (pH 6.0) or 20 mM HEPES-NaOH (pH 7.0 and pH 8.0). Closed symbols, KEA3; open symbols, pPAB404. Error bars indicate SD (n = 3).

Although KEA1-KEA6 are classified as cation-proton antiporters^12^, there is no direct evidence of their antiport activity. We tested the transport function of KEA1-KEA6 through heterologous expression in *E*. *coli* (Figs 1–3). K^+^/H^+^ or Na^+^/H^+^ antiport activity of KEA1-KEA6 was monitored by measuring the increase of acridine orange fluorescence after addition of KCl (Fig. 3a) or NaCl (Fig. 3b) at pH 8 to everted membrane vesicles isolated from TO114 transformants. Due to K^+^/H^+^ or Na^+^/H^+^ antiport activity, H^+^ efflux from everted vesicles leads to an increase of the pH inside the vesicles which in turn results in dequenching. Addition of NaCl to membrane vesicles containing the positive control NhaS3 caused an increase in fluorescence^23^, whereas addition of KCl or NaCl did not result in obvious fluorescence dequenching in vesicles containing KEA1-KE6. They did not show clear cation-proton antiporter activities. The properties of KEA1-KEA6 obtained in the above are summarized in Fig. 3c.

KEA3 is the closest homolog to *Synechocystis* NhaS3, and both are present in the thylakoid membrane. Because of these similarities, we focused on KEA3 to further investigate the structure and function of KEAs. KEA3 has three isoforms, KEA3.1, KEA3.2 and KEA3.3, due to alternative splicing (see below). When KEA3.1 and KEA3.3 were analyzed in the same way as described above for KEA3.2, no K^+^/H^+^ or Na^+^/H^+^ activity was detected (Supplementary Fig. S2a,b). Since results from previous of genetic and physiological experiments suggested that KEA3 functions as a K^+^/H^+^ antiporter^10,14,15,17^, we tested the effect of pH ranging from 6.0 to 8.0 on the K^+^ export activity of KEA3 (Fig. 3d). However, no significant pH dependence was detected. The N-terminal region of KEA3 likely affects its membrane integration^18^. Therefore, we generated a chimeric KEA3 which has the N-terminal cytoplasmic region of NhaS3 replacing the corresponding sequence of KEA3 (Supplementary Fig. S2c,d). The chimeric protein also did not show any K^+^/H^+^ or Na^+^/H^+^ antiport activity. This lack of measurable cation/H^+^ antiport activity of KEA3 is consistent with the lack of pH dependence of K^+^ efflux activity of KEA3 (Fig. 3d) and the failure of KEA3 to rescue the loss of Na^+^/H^+^ antiporter function of the *E*. *coli* TO114 strain (Fig. 2a). For our initial experiments investigating the antiport activity of KEA3 we used pPAB404 as vector. We also tested another vector, pTrcHis2B (Thermo Fisher Scientific), which contains a translational enhancer^30^ and minicistron^31^ to increase the translational efficiency for KEA3 expression (Supplementary Fig. S2e). However, even then no antiporter activity was detected.

### Examination of the K^+^ and Na^+^ selective filter in KEA3

**Figure 4:**
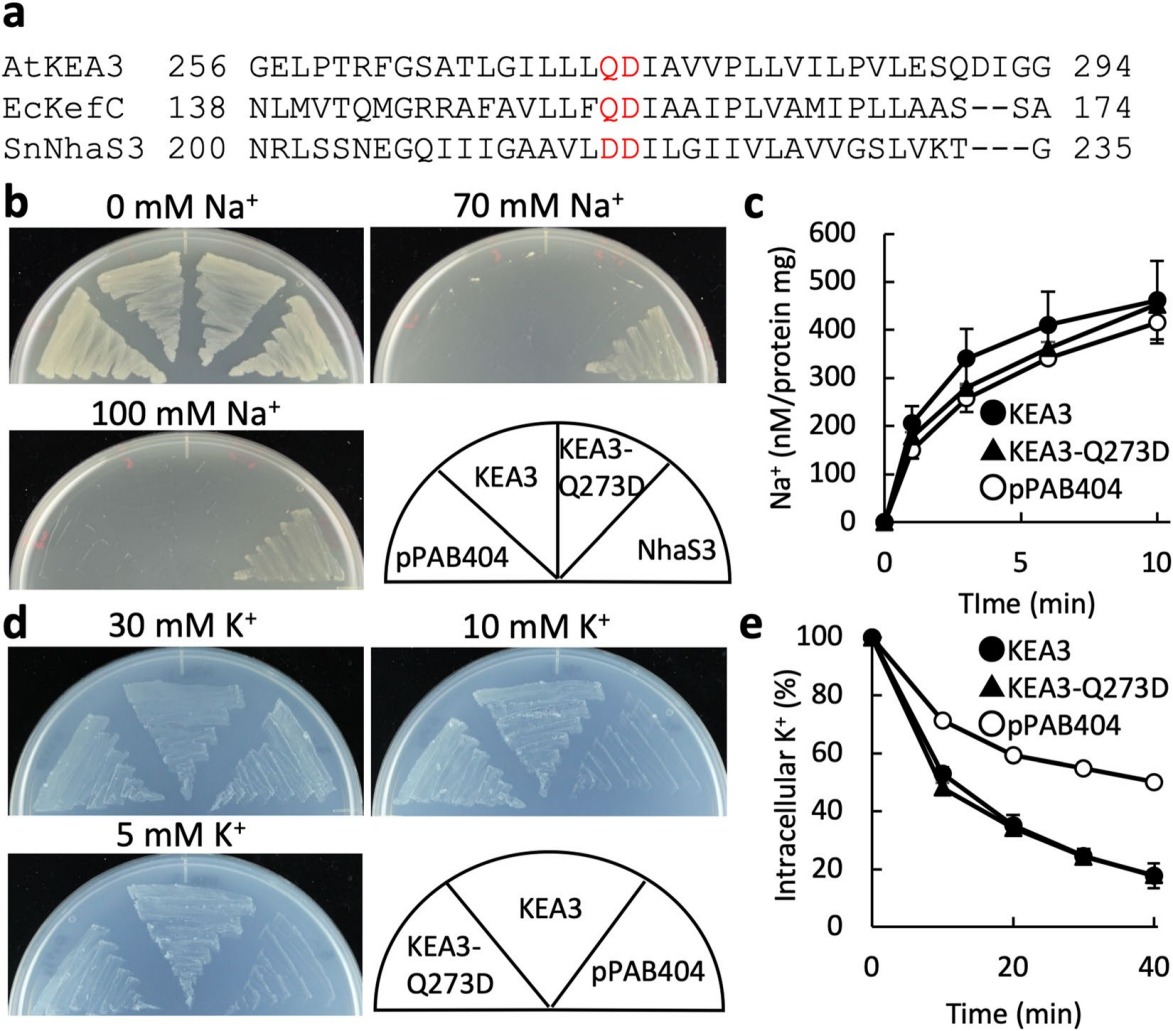
Ion transport activity of KEA3-Q273D. (**a**) Amino acid alignment of Arabidopsis KEA3, *E*. *coli* KefC (EcKefC) and *Synechocystis* NhaS3. The residues that may determine selectivity of K^+^ and Na^+^ are shown in red. (**b**) Salinity tolerance of *E*. *coli* TO114 containing KEA3-Q273D, KEA3, NhaS3 or pPAB404 on media supplemented with 0, 70 or 100 mM NaCl at 30 °C for 24 h. (**c**) Na^+^ uptake activity of *E*. *coli* TO114 containing KEA3, KEA3-Q273D or the empty vector. Na^+^ influx was initiated by addition of 10 mM NaCl (final concentration) into the buffer at t = 0. Error bars indicate SD (n = 3). (**d**) Growth assay of *E*. *coli* LB2003 containing KEA3-Q273D, KEA3 or pPAB404. Cells were grown on synthetic medium supplemented with 5, 10 or 30 mM KCl at 30 °C for 24 h. (**e**) K^+^ efflux rate from the same *E*. *coli* TO114 transformants as in (**c**). Cells (16 μL) were added into 200 μL of a solution containing 0.4 M NaCl and 20 mM HEPES-NaOH (pH 8.0) at t = 0. Error bars indicate SD (n = 3).

Na^+^/H^+^ antiporters have conserved double aspartates (DD) in TM5, which serve as Na^+^ and H^+^ binding sites^32,33^. KEA1-KEA6 and other K^+^/H^+^ antiporters have a glutamine (Q) or asparagine (N) instead of the first aspartate of the DD motif (Fig. 4a). It has been postulated that the Q/N is a determinant for K^+^ selectivity^24^. We tested whether replacing Q with D has an effect on Na^+^/H^+^ antiport activity of KEA3 (KEA3-Q273D). *Synechocystis* NhaS3, which is the closest homolog of KEA3, possesses the DD motif and showed strong fluorescent quenching after NaCl addition^23^ (Fig. 3b). In contrast, KEA3-Q273D showed neither Na^+^/H^+^ nor K^+^/H^+^ exchange activity (Supplementary Fig. S2f). Moreover, Na^+^ efflux and Na^+^ uptake activity of KEA3-Q273D were not significantly different from those of the wild-type KEA3 (Fig. 4b,c). On the other hand, KEA3-Q273D complemented the K^+^-uptake deficient LB2003 strain at low KCl (5 and 10 mM) (Fig. 4d) and extruded K^+^ as well as the wild-type KEA3 (Fig. 4e). These results indicate that a Q in the first position of the DD motif is not involved in determining the ion selectivity of K^+^ over Na^+^ in KEA3.

### Two splice variants, KEA3.1 and KEA3.3, showed decreased activity of K^+^ transport

**Figure 5:**
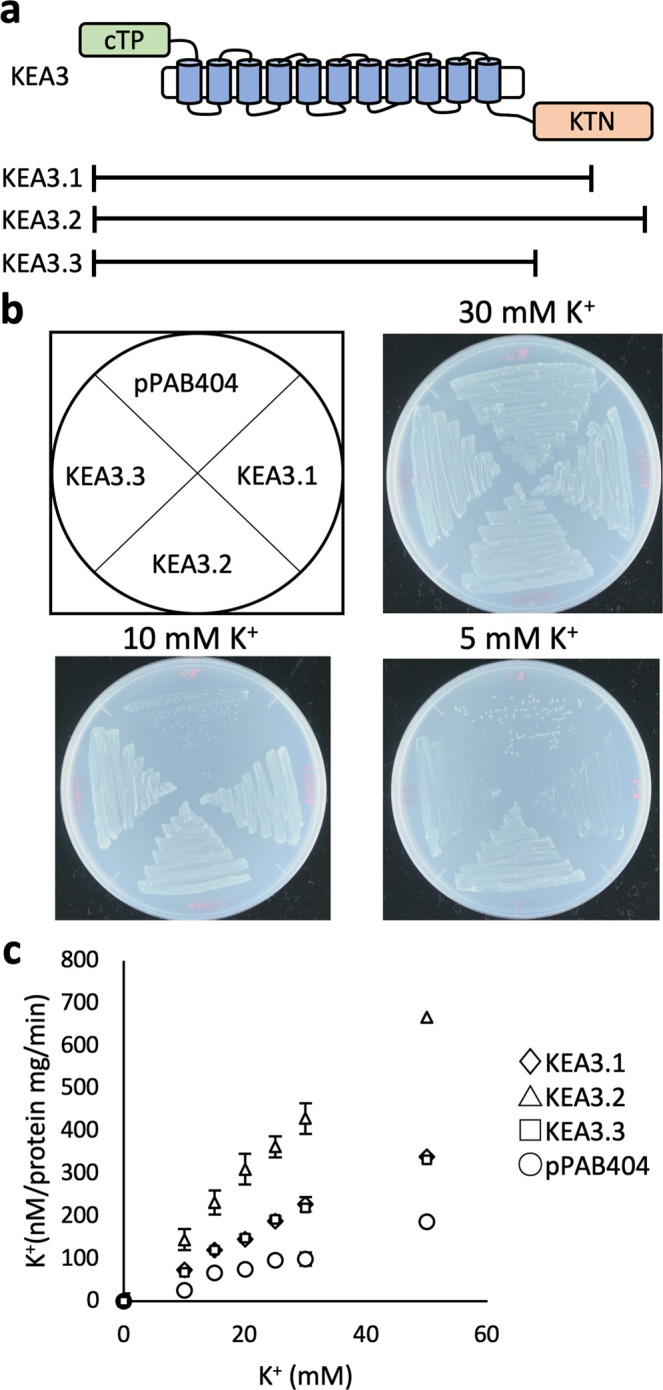
K^+^ uptake activities of KEA3 splice variants. (**a**) Diagram depicting the three splice variants KEA3.1, KEA3.2 and KEA3.3. cTP, chloroplast transit peptide; KTN, K^+^ transport nucleotide binding domain. (**b**) Growth of *E*. *coli* LB2003 containing FLAG-KEA3.1, 3.2, 3.3 or the empty vector on synthetic medium supplemented with 5, 10 or 30 mM KCl at 30 °C for 24 h. (**c**) K^+^ content of the same transformants as in (**b**) incubated in 200 mM HEPES-NaOH (pH 8.0) with varying concentrations of KCl. Aliquots were taken after incubation for 1 min. Error bars indicate SD (n = 3).

**Figure 6:**
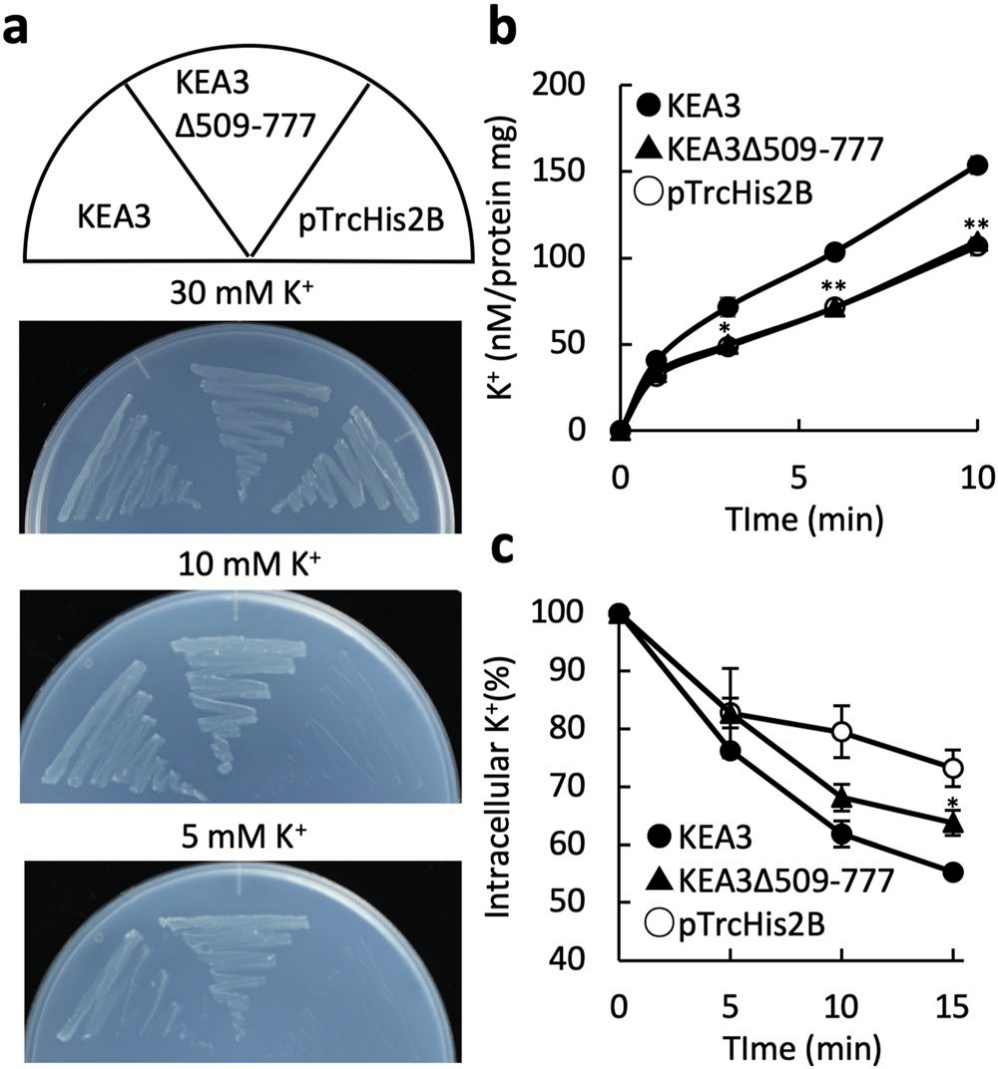
K^+^ transport activities of KEA3 variants lacking the KTN domain. (**a**) Growth of *E*. *coli* LB2003 containing KEA3, KEA3∆509-777 or the empty vector pTrcHis2B on synthetic medium supplemented with 5, 10 or 30 mM KCl at 30 °C for 24 h. (**b**) K^+^ uptake activity by the same *E*. *coli* LB2003 transformants. KCl was added to the cell suspension to a final concentration of 10 mM in 200 mM HEPES-NaOH (pH 8.0). Error bars indicate SD (**p < 0.01, 0.01 < *p < 0.05, n = 3). (**c**) K^+^ efflux from *E*. *coli* TO114 containing pTrcHis2B-KEA3, pTrcHis2B-KEA3∆509-777 or pTrcHis2B. The cells (16 μL) were added to 200 μL of 0.4 M NaCl and 20 mM HEPES-NaOH (pH 8.0) at t = 0. Error bars indicate SD (*p < 0.05, n = 3).

Three different splice variants of *KEA3* have been identified (Fig. 5a)^17^. KEA3.2 is the most abundant isoform in Arabidopsis and was used for the experiments in Figs 1–4^17^. In *KEA3*.*1* the 16th intron is not spliced out, resulting in a frame shift. In consequence, KEA 3.1 is a shorter protein that is missing 153 residues from its C-terminal region, compared with KEA 3.2. KEA3.3 is a minor variant that is lacking the KTN domain (Fig. 5a)^17^. We compared the cation transport function of all three variants (Fig. 5b,c). LB2003 cells expressing KEA3.1 and KEA3.3 were able to grow in medium containing 10 mM KCl, but not 5 mM KCl (Fig. 5b). The K^+^ uptake rate of KEA3.1 and KEA3.3 was lower than that of KEA3.2 (Fig. 5c). As for KEA3.2, no Na^+^/H^+^ and K^+^/H^+^ antiport activity could be detected for KEA3.1 and KEA3.3 (Supplementary Fig. S2a,b). The low activity of KEA3.1 and KEA3.3 may be due to their lack of a KTN domain. To corroborate this, we inserted a stop codon between TM11 and the KTN domain in the *KEA3*.*2* sequence and introduced the construct (KEA3∆509-777) into both *E*. *coli* mutants (Fig. 6, Supplementary Fig. S1). KEA3∆509-777 was able to support the growth of the potassium uptake-deficient strain LB2003 in media containing 5 mM KCl (Fig. 6a). However, KEA3∆509-777 showed decreased K^+^ influx and efflux, compared with KEA3.2 (Fig. 6b,c). This indicates that the KTN domain has a role in supporting KEA3-mediated cation transport.

### Assessment of KEA3-G422R discovered in the in the Arabidopsis *dpgr* mutant

**Figure 7:**
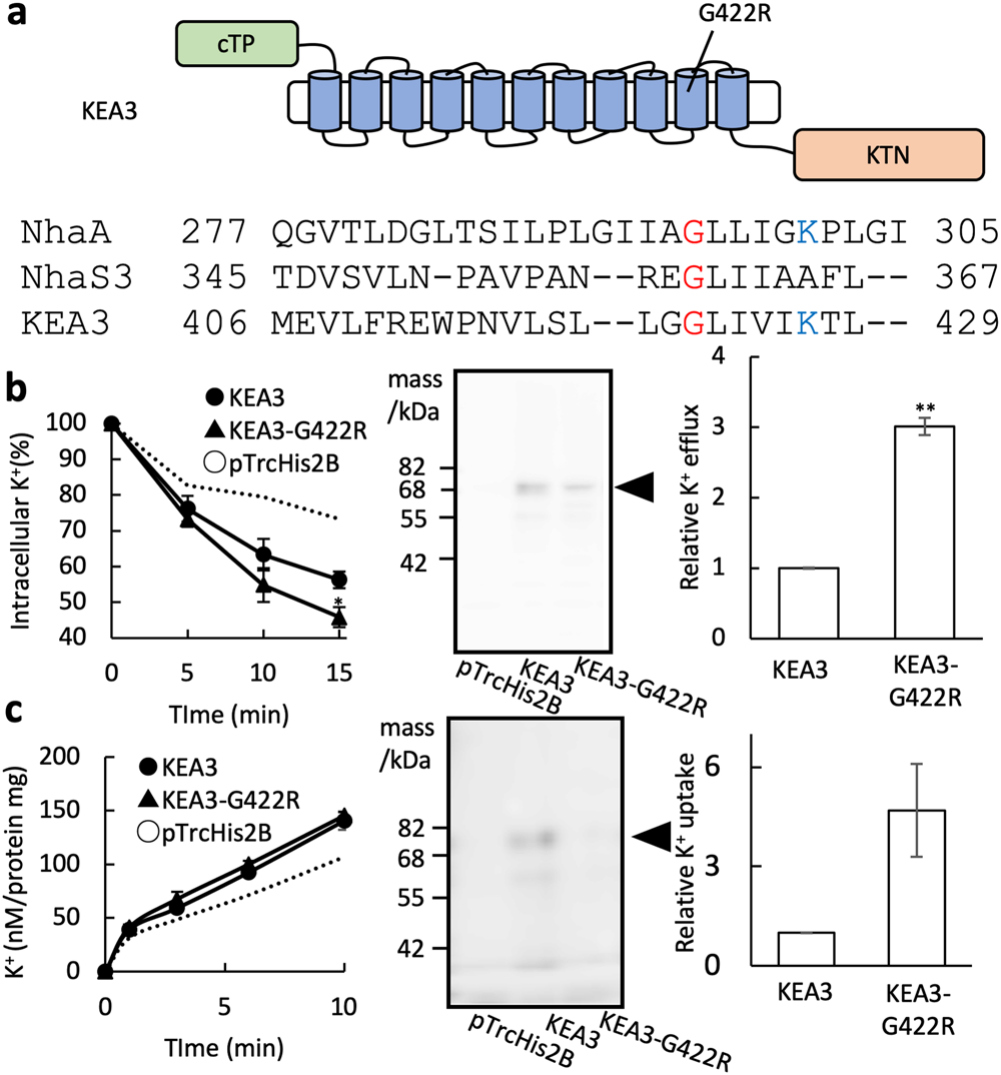
Assessment of KEA3-G422R variant function. (**a**) Position of the Arabidopsis *dpgr* point mutation (G422R) in the 10th transmembrane domain in KEA3 (top) and amino acid sequence alignment showing the corresponding region of *E*. *coli* NhaA, *Synechocystis* NhaS3 and Arabidopsis KEA3 (bottom). The conserved Gly (G422 in KEA3) and the Lys that is involved in ion transport are marked in red and blue, respectively. (**b**) K^+^ efflux measurement of *E*. *coli* TO114 containing KEA3-His-tag, KEA3-G422R-His-tag or the empty vector pTrcHis2B. Cells (16 μL) were mixed with 200 μL of 0.4 M NaCl in 20 mM HEPES-NaOH (pH 8.0) at t = 0. Error bars indicate SD (*p < 0.05, n = 3). The broken line represents the same data for *E*. *coli* containing the empty vector pTrcHis2B shown in Fig. 6c for comparison (left). Representative profile of immunoblot detection of KEA3-His-tag and KEA3-G422R-His-tag proteins. Membrane fractions corresponding to 7 μg of protein per lane were loaded onto the gel. Quantification of bands was conducted with ImageJ. Full images of representative data are indicated in Supplemental Fig. S3. The arrow is pointing at the KEA3/KEA3-G422R band (center). Relative K^+^ efflux activity of KEA3-G422R-His-tag with respect to KEA3-His-tag was calculated from the data at 15 min in the left panel (**p < 0.01, n = 3) (right). (**c**) K^+^ uptake by *E*. *coli* LB2003 containing KEA3-His-tag, KEA3-G422R-His-tag or pTrcHis2B. KCl was added to the cells to a final concentration of 20 mM (pH 8.0). Error bars indicate SD (n = 3). The broken line represents the same data for *E*. *coli* containing pTrcHis2B shown in Fig. 6b for comparison (left). Representative profile of immunoblot detection of KEA3-His-tag and KEA3-G422R-His-tag. Membrane fractions corresponding to 7 μg of protein were loaded onto the gel. Quantification of proteins was conducted with ImageJ (center). Full images of representative data are indicated in Supplemental Fig. S3. Relative K^+^ uptake activity of KEA3-G422R-His-tag with respect to KEA3-His-tag was calculated from the data at 10 minutes in the left panel. Error bars indicate SD (n = 3) (right).

Wang *et al*. (2017) reported the isolation of the Arabidopsis *dpgr* (*disturbed proton gradient regulation*) mutant, which contains a point mutation converting the Gly at position 422 in KEA3 to Arg^15^. In the *dpgr* mutant, which contains a dominant allele of *KEA3*, transient induction of NPQ was inhibited during induction of photosynthesis^15^. The change of Gly to Arg (G422R) might interfere with the down-regulation of K^+^ transport activity of KEA3. For the determination of the K^+^ transport activity of KEA3, we used versions of KEA3.2 and KEA3-G422R that contained a 6xHis-tag fused to their C-termini which enabled detection by immunoblot (Fig. 7b,c). Remarkably, KEA3-G422R exhibited a 3.0-fold increase in relative K^+^ efflux activity (Fig. 7b) and a 4.7-fold increase (Fig. 7c) in relative K^+^ influx activity, compared to the wild type. These results indicate that the *dpgr* mutation enhances KEA3 activity.

## Discussion

This study is the first direct comprehensive assessment of KEA1-KEA6 transport activity. Based on the results of several physiological experiments in combination with genetic studies of Arabidopsis mutants, KEA1-KEA6 have been categorized as K^+^/H^+^ antiporters^10,14–20,34^. Direct evidence for their ion transport activity is still lacking, suggesting that it is difficult to measure. Considering the endosomal localization of KEA1-KEA6 in Arabidopsis^35^, we used an *E*. *coli* expression system to evaluate their transport function^26^. This enabled us to detect both K^+^ influx and efflux transport activity. KEA1-6 were selective for K^+^ over Na^+^, indicating that KEA1- KEA6 were involved in ∆Ψ formation. Unexpectedly, antiport activity could not be detected for KEA1-KEA6 (Fig. 3 and Supplementary Fig. S2).

The light reactions of photosynthesis are accompanied by a slight swelling of the thylakoids due to the influx of H^+^ into the thylakoid lumen^36^, and carbon assimilation is expected to cause an increase in osmolarity in the chloroplast. K^+^ is a major cation closely involved in the regulation of intracellular and chloroplast osmolarity^37^. Therefore, translocation of cations across the chloroplast envelope and the thylakoid membrane directly or indirectly affects chloroplast osmolarity. The KEA homolog, *Synechocystis* NhaS3 plays an important role in the response to changes in osmotic pressure of the external solution^23^. A similar physiological role has been proposed for KEA1-KEA3 by Stephan *et al*.^19^. KEA1-KEA3 in plastids may play an important role in the initial rapid Ca^2+^ response to hyperosmotic stimuli. The *kea1 kea2* double and the *kea1 kea2 kea3* triple mutants have swollen chloroplasts and the plastid envelope is disrupted in the triple mutant^10^. The aberrant shape of the chloroplasts in the knockout mutants likely reflects a dysfunction of the osmotic response in the chloroplasts. An absence of KEA1-KEA3-mediated K^+^ extrusion from the stroma in the mutants would lead to increased water entry into the chloroplast. The defects of KEA1 and KEA2 in the *kea1 kea2* mutants can be rescued by exogenous NaCl addition^10^. Therefore, sequestration of K^+^ in the thylakoid lumen due to the activity of KEA3 might help prevent the swelling of the chloroplasts during the light reaction (Figs 1, 4–7). In addition, it has been reported that another triple mutant (*kea4 kea5 kea6*) is sensitive to high salinity^20^, therefore KEA4-KEA6-mediated K^+^ translocation (Fig. 1) may be involved in protecting organelles from osmotic rupture, similar to the role of NhaS3 in *Synechocystis*.

The KTN domain, located in the C-terminal region of KEA1-KEA3 is known as the regulator of K^+^ conductance (RCK) domain in the bacterial Ca^2+^-activated K^+^ channel, MthK. The RCK domain in the C-terminal region of MthK requires mutual interaction of each channel subunit for K^+^ transport activity^38^. The K^+^ uptake transporter, KtrB in *Vibrio alginolyticus* recruits the ancillary cytosolic subunit, KtrA, which binds to ATP^39^. The *E*. *coli* K^+^/H^+^ antiporter, KefC, contains a KTN domain in its C-terminal region. The KTN domain suppresses KefC transport activity when glutathione and NADH bind to the KTN^24,25^. Based on these features of KTN/RCK, it is possible that the KTN domain in KEA3 might serve as a nucleotide-binding site for NADP(H) or ADP/ATP, which may control gating of K^+^ conducting pore^10,12,13,15,17^. Two short splice variants of KEA3, KEA3.1 and KEA3.3, showed lower K^+^ transport activity, compared to the full-length KEA3.2 (Fig. 5). Similarly, deletion of the KTN domain resulted in a strong decrease in K^+^ influx and efflux activity (Fig. 6). These results suggest that the KTN domain might participate in the regulation of K^+^ transport activity in KEA3^14,15,17^. In KefC, KTN contains a regulatory loop comprising the HALSDIEP sequence, which is involved in transport regulation via glutathione^40,41^. As shown in Supplementary Fig. S1, the regulatory loop is less conserved in KEA3. A structure analysis identified several residues responsible for glutathione binding in the KTN domain of KefC^25^. These residues are also less well conserved in KEA3 (Supplementary Fig. S1). These differences in amino acid sequence imply that the function of the KTN domain in KEA3 may be different from that in KefC. KEA4-KEA6 do not contain a KTN domain (Fig. 1 and Supplementary Fig. S1). Due to low homology between the KTN domains of KEA1-KEA3 and KefC, and the absence of the KTN domain from KEA4-KEA6, we used the *E*. *coli* TO114 mutant instead of the *kefB kefC* mutant, although previously it had been shown that the *kefB kefC* mutant could be complemented by KEA4-KEA6 when using glutathione-mediated activation via the KTN domain^20^.

The DD motif is critical for the cation transport activity of the NhaS3. This was confirmed by the finding that the conversion of the DD motif to AA in NhaS3 abolishes its antiporter activity^23^. It has been suggested that a D in the first position is required for Na^+^/H^+^ antiport activity while N/Q instead of the first D is required for K^+^ translocation, based on a comparison analysis of the amino acid sequence and cation transport properties of many Na^+^/H^+^ or K^+^/H^+^ antiporters^14^. More recently, it has been proposed that the second D in the motif is responsible for binding and translocation of a H^+^ and a K^+^ (or Na^+^) in AtCHX17, which has ND in the motif^11^. Our data in Fig. 4 support the latter prediction since replacement of Q with D at position 273 in KEA3 did not give rise to Na^+^ transport activity and did not affect K^+^ translocation property. Based on a large phylogenetic comparison KefB and KefC, K^+^/H^+^ antiporters from *E*. *coli*, mentioned above, are close homologs of KEA3^42^. The transmembrane domain of KefC shares 33% identity with that of KEA3, and 25% with that of NhaS3. Another region besides the DD motif may be involved in K^+^ and Na^+^ translocation in the KEAs although it can not be ruled out the possible contribution of the DD motif to the ion selectivity.

During induction of photosynthesis, knockout of the *KEA3* gene leads to a delay in the relaxation of NPQ. However, the Arabidopsis *dpgr* mutant, which carries a point mutation in KEA3 (G422R), has a dominant phenotype with impaired induction of transient NPQ^15^. Consistent with this observation, KEA3-G422R showed enhanced K^+^ transport activity (Fig. 7). The corresponding Gly is not found in other KEAs but it is conserved in *Synechocystis* NhaS3 (G360) and *E*. *coli* NhaA (G296) (Fig. 7b, Supplementary Fig. S1). Analysis of the crystal structure indicates that a conserved Lys (K300 in NhaA and K305 in NapA), adjacent to the second conserved Asp in the DD motif^32,33^, participates in a pH-dependent activation mechanism^32,33^. KEA3 has a corresponding Lys (K427) in the vicinity of G422 in TM10. Introduction of the positively charged Arg instead of G422 might increase the amplitude of K^+^ transport in KEA3 by affecting the local environment around the pH sensing sites.

The *kea3* knockout mutant showed an increase in ΔpH^10,14,16^. The previous reports showed that NhaS3, which is homologous to KEA3, likely dissipates ΔpH by transporting H^+^ to the cytoplasm and Na^+^ to the thylakoid lumen, thereby preventing an over-accumulation of H^+^ in the thylakoid lumen in the light and/or during salt stress-induced inactivation of photosystem II in *Synechocystis*^23,43^. Likewise, it has been proposed that conversion of ΔpH to ∆Ψ occurs due to K^+^ and H^+^ exchange by KEA3 across the thylakoid membrane^14,16^. This study showed that KEA3 functioned as K^+^ efflux system. KEA3 can load K^+^ into the thylakoid lumen (Figs 1, 4–7). Simultaneously, it would push out H^+^ from the lumen to the stroma, leading to lower ΔpH. Therefore, K^+^ efflux activity of KEA3 is crucial for ΔpH dissipation in the thylakoid membrane. Enhanced K^+^ transport activity from stroma to lumen mediated by KEA3-G422R would reduce ΔpH, leading to repression of NPQ in chloroplasts.

K^+^/H^+^ transport coupling in KEA3 was not obtained; despite several attempts including replacement of vector (Fig. 6 and Supplementary Fig. S2e), swapping of N-terminal region (Supplementary Fig. S2c,d), conversion of Q to D (Fig. 4), and testing of pH dependency of K^+^ passage (Fig. 3d). KEA3 exhibited K^+^ translocation activity and NhaS3 shows reproducibly significant Na^+^/H^+^ antiport activity in the *E*. *coli* assay system used in our study. There are a number of reasons why K^+^/H^+^ antiporter activity was not detected for KEA1-KEA6, even though they may have this activity in Arabidopsis. First, KEAs may require some accessory components for coupling K^+^ transport activity of KEAs with H^+^ counter-directed movement, which was missing in our *E*. *coli* expression system. *E*. *coli* K^+^/H^+^ antiporters, KefB and KefC require auxiliary proteins for their full activity^44^. Second, the *E*. *coli* membrane may not be a perfect fit for the amino acid arrangement in the KEAs’ ion conduction pore to enable the coupling of K^+^ and H^+^. Third, it may be possible that the H^+^ transport activity was too small to be detected in our system (Fig. 3).

In summary, our comprehensive examination of Arabidopsis KEA1-KEA6 function has shown that KEA1-KEA6 act as a K^+^ transport system. The transport function of KEA3 was not completely identical to that of NhaS3 with respect to its cation selectivity and antiporter activity^23^. Nevertheless, KEA3 and NhaS3 likely share the same physiological role in osmoadaptation to maintain chloroplast integrity and promote dissipation of ΔpH in the thylakoid membrane.

## Methods

### Plasmid construction

All primers used in this study are listed in Supplementary Table 1. The entire cDNA of AtKEA3 was constructed from two different cDNA products listed in the full-length cDNA library (RIKEN GSC) by PCR using primers PPAB404-BamHI-KEA3-F and KEA3-488bp-R and KEA3-473bp-F and KEA3-BamHI-PPAB404-R. The PCR fragments were inserted into *Bam*HI site of pPAB404^45^ with the SLICE method^46^. The resultant plasmid contained a point mutation in the *KEA3* sequence. The mutated position was replaced by PCR-amplification with a pair of primers, KEA3-1157bp-F and KEA3-1156bp and self-ligation. The resultant plasmid was designated as pPAB404-KEA3.1. For *KEA3*.*2* construction, the sequence corresponding to the 16th intron was removed from pPAB404-KEA3.1 by PCR-amplification with a pair of phosphorylated primers, KEA3-1869bp-R and KEA3-1870bp-F and self-ligation. The resultant plasmid was designated as pPAB404-KEA3.2. To create pPAB404-KEA3.3, AAAATAGGT was eliminated from pPAB404-KEA3.2 by PCR-amplification with a pair of primers, KEA3-1539bp-R and KEA3-1547bp-F and self-ligation. The chloroplast transit peptide of KEA3 was removed by PCR amplification with primers ATG-KEA3-201bpF and PPAB-BamHI-Rv. The clones encoding KEA3-G422R and KEA3-Q273D variants were constructed by PCR-amplification with a pair of primers, KEA3dpgrF and KEA3dpgrR, and Q273DR and Q273DF with pPAB404-KEA3.2 as template. To add a FLAG-tag at the N-terminus and a His-tag at the C-terminus of KEA3 pPAB404-KEA3.2 was amplified using primers Flag-KEA3-F-201bp and Flag-ATG-KEA3-R (FLAG-KEA3) or KEA3-HIS-1219-Forward and KEA3-HIS-1219-REVERSE (KEA3-His). To replace the N-terminal region of KEA3 (amino acids 1–90) with the NhaS3 N-terminal region (amino acids 1–25), each piece was amplified separately by PCR using primers ppabbamHiNhaS3_0920 and KEA3_NhaS3_0920 (NhaS3 N-term) or NhaS3-KEA3_0920 and KEA3-BamHI-PPab404-R (KEA3∆1-90), with either genomic DNA of *Synechocystis* sp. PCC6803 or pPAB404-KEA3.2 as template. The PCR fragments were ligated into the *Bam*HI site of pPAB404 with the SLICE method. His-tagged KEA3 isoforms were obtained from pPAB404-KEA3.2-His digested with *Kpn*I and *Xba*I and ligated into the *Kpn*I-*Xba*I sites of pTrcHis2B. To isolate cDNAs encoding KEA1, KEA2, KEA4, KEA5 and KEA6, total RNA was extracted from tissue of *Arabidopsis thaliana* Col-0 followed by DNase treatment. The reverse transcription reaction was performed using ReverTraAce qPCR RT Master Mix with gDNA remover (TOYOBO), and each cDNA was obtained by PCR-amplification with the primers listed in Supplementary Table 1. All PCR products were ligated into the *Bam*HI site in pPAB404 with the SLICE method.

### Measurement of K^+^/Na^+^ uptake in *E*. *coli*

K^+^ influx into *E*. *coli* LB2003 (F-, *thi*, *lacz*, *gal*, *rha*, ∆*kdpFABC5*, *trkD1*, ∆*trkA*) containing the empty vector or KEA1-KEA6 was measured as described previously^47^. Briefly, *E*. *coli* were cultured in synthetic medium supplemented with 30 µg/ml ampicillin, 30 mM KCl and 100 µM isopropyl *β*-D-1-thiogalactopryanoside (IPTG) at 30 °C. Cells were harvested and suspended in 120 mM Tris-HCl (pH 8.0) and 1 mM EDTA-NaOH (pH 8.0) to an OD_578_ of 30. After incubation for 30 min at 37 °C, cells were collected by centrifugation and washed three times with 200 mM HEPES-NaOH (pH 8.0). After shaking at room temperature for 20 min, the concentration of the cell suspension was adjusted to an OD_578_ of 3.0 with the same buffer. For the experiments, glucose was added to a final concentration of 10 mM prior to KCl addition (final concentration of 10 mM) (t = −10 min). Samples (1 ml) were taken at the indicated times followed by centrifugation through silicon oil. The collected cells were disrupted by addition of 5% trichloroacetic acid at 100 °C for 5 min. After centrifugation, the pellets were used for BCA protein assay^48^ and the K^+^ content of the supernatant was determined using an atomic absorption spectrometer. For Na^+^ uptake measurements, *E*. *coli* TO114 (W3110 *nhaA*::Km^r^ *nhaB*::Em^r^ *chaA*::Cm^r^) was used as the host strain. The strain was grown in KLB medium supplemented with 50 μg/ml of ampicillin and 250 µM of IPTG. Na^+^ influx was measured as described above for K^+^ uptake except HEPES-TEA (pH 8.0) was used instead of HEPES-NaOH.

### K^+^ (Na^+^)/H^+^ antiporter assay

Everted membrane vesicles of *E*. *coli* TO114 were prepared as described previously^49^. Membrane vesicles equal to 20 µg of protein were added into buffer containing 10 mM Tris-HCl (pH 8.0), 140 mM choline chloride, 5 mM MgSO_4_ and 1 μM acridine orange. Tris/D-lactate (10 mM) was added to initiate fluorescence quenching by respiration. Dequenching of fluorescence was monitored after the addition of 8 mM KCl or NaCl. Then NH_4_Cl (final concentration: 25 mM) was added to dissipate the pH gradient across everted membranes. Fluorescence emission was monitored at 525 nm with excitation at 492 nm.

### Measurements of K^+^ efflux

K^+^ efflux was measured as described previously^50^ with minor modifications. *E*. *coli* TO114 was grown in LBK300 (pH7.5) supplemented with 50 µg/ml of ampicillin and 250 µM of IPTG. When the cell culture reached OD_660_ = 0.6–0.8, cells were harvested, washed with 0.4 M KCl, and resuspended in 0.4 M KCl. A 16 µL aliquot of cells was added to 200 µL of reaction buffer containing 20 mM HEPES-NaOH (pH 8.0) and 0.4 M NaCl. After incubation for the indicated time, cells were collected by centrifugation through silicone oil. K^+^ content of the cell pellets was determined as described above.

### Immuno-detection of membrane proteins

*E*. *coli* LB2003 cells were collected by centrifugation and washed with 500 mM NaCl in 50 mM Tris-HCl (pH 7.0). Cells were disrupted with a French press, followed by centrifugation at 15,000 g for 15 min. Supernatant was collected and the membrane fraction was harvested by ultracentrifugation at 250,000 g for 30 minutes. Pellets were resuspended with 600 mM NaCl and 10% glycerol in 50 mM Tris-Cl (pH 8.0). Samples were stored in −80 °C. For immunodetection, for each sample 7 µg of protein were separated by electrophoresis on a SDS-polyacrylamide gel (10%) and transferred electrophoretically onto 0.45 µm PVDF membrane. Immunodetection of His-tagged proteins was conducted with Anti-His-tag mAb-HRP-DirecT (MBL) and ECL Western blotting detection reagents (GE Healthcare UK Ltd).

## Supplementary information

Supplemental table, Supplemental figure1, Supplemental figure2, Supplemental figure3
