# Supplementary material for: Evidence for potassium transport activity of Arabidopsis KEA1-KEA6
Source: Sci Rep. 2019 Jul 11;9:10040. doi: 10.1038/s41598-019-46463-7 (PMC6624313; doi:10.1038/s41598-019-46463-7)
Supplement: Supplementary file 1 — Supplemental table, Supplemental figure1, Supplemental figure2, Supplemental figure3 [file 41598_2019_46463_MOESM1_ESM.pdf]

Title: Evidence for potassium transport activity of Arabidopsis KEA1-KEA6

Author: Masaru Tsujii, Kota Kera, Shin Hamamoto, Takashi Kuromori, Toshiharu Shikanai, Nobuyuki Uozumi

This file includes Supplemental figure S1-Supplemental figure S3 and Supplemental Table1.

Supplementary Table 1. Primers used in this study

Table 1 List of primers used to prepare plasmids for expression of KEAs in *E. coli*

Name	Sequence (5' to 3')
PPab404-BamHI-KEA3-F	CGGTACCCGGGGATCATGGCAATTAGTACTATGTTAGGGTCCAT
KEA3-488bp-R	AGGAAAAGAATCCCCCATTCTGAAAGAAC
KEA3-473bp-F	GGGGATTCTTTTCCTGCTCTTTGAGATGGGTCTCGAGC
KEA3-BamHI-PPAB404-R	CGACTCTAGAGGATCTTAATCTTGAGCTTTATCAGCTTTACCAAC
KEA3-1157bp-F	GGCCATTTAGAGGTTTACTTCTC
KEA3-1156bp-R	TAATATCAGCTTCAATTTGTGTTCGGAAATTTG
KEA3-1869bp-R	CCCGGGGAAGGCAAGACGG
KEA3-1870bp-F	AGTCCTATTTACGCAAGAGCTCAAGATTTAC
KEA3-1539bp-R	CTCTCCGGGATCAAGTCTCTCATC
KEA3-1547bp-F	GTGAAGATGTGAACTTCGATGTTAG
KEA3dpgrF	CGTCTGATTGTGATCAAAACACTGATTATAACC
KEA3dpgrR	ACCCAAGAGTGAGAGGACATTAGGC
Q273DR	ATCTAGAAGAAGAATTCCCAAGGTTGCTGAGCCAAATC
Q273DF	GATATAGCTGTTGTGCCGTTACTAGTCATTCTTCCAGTG
KEA3-His1219F	CATCACCATCACCATCACTAAGATCCTCTAGAGTCGACCTGCAG
KEA3-His1219R	ATCTTGAGCTTTATCAGCTTTACCAACAAATCC
ATG-KEA3-201bpF	ATGACCTCTAAGAGATTCTATTTCCAAGGG
PPAB-BamHI-Rv	GATCCCCGGGTACCGAGCTC
Flag-KEA3-F-201bp	GATGATGATAAAACCTCTAAGAGATTCTATTTCCAAGGG
Flag-ATG-KEA3-R	ATCTTTATAATCCATGATCCCCGGGTACCGAGC
ppabbamHiNhaS3_0920	CGGTACCCGGGGATCATGTTTATGAACCCATTGCTCCCTCCC
KEA3_NhaS3_0920	CGCTACATCTACTCCAGCTATTTCCGTTTCTGTTTCTACGGC
NhaS3-KEA3_0920	GGAGTAGATGTAGCGAGTGCTGTTG
ppab404_ATG_KEA1	CGGTACCCGGGGATCATGAAGAGAATTAAGAAACTACTTGAGATGATCC
ppabbamHiKEA1	CGACTCTAGAGGATCTCAGATTACGACTGTGCCTCCTTCG
ppab404_ATG_KEA2	CGGTACCCGGGGATCATGAAGAGAATAAAGAAACTACTTGAGATGTTCC
ppabbamhiKEA2	CGACTCTAGAGGATCTTAGATAGCGAGTGTGCCTTCAATAATCTG
ppab404_ATG_KEA4	CGGTACCCGGGGATCATGCGGCGGTGTAAAAACAACACTGAC
ppabbamhiKEA4	CGACTCTAGAGGATCTCAAGAGTCGTGAAGAGAACCTTGGATC

ppab404_ATG_KEA5	CGGTACCCGGGGATCATGGCGAGATTCGCAGTGATTGGATTAAC
ppabbamhiKEA5	CGACTCTAGAGGATCTCACTTGGTTCTGTTATGTACTTCTATCAATG
ppab404_ATG_KEA6	CGGTACCCGGGGATCATGGTGGAAGGAAGAAGAAGAAGAAGATTC
ppabbamhiKEA6	CGACTCTAGAGGATCTTAGGAGCTGTGGGATTGACGTGACATTAG

```
KEA6     1                                                                                         MVEGRR     6

KEA4     1   MRRCKNNTDKFSVITMRLLTLLLICTFFFFFSFAYSAESDNETDSVVTREINGTVVESNATSAKPREDSFADMIDRALEKEFPDNDQN   88
KEA5     1              MARFAVIGLTFLLLLGTSLS-ARSDEETRERFYGNVVNSTAPGNGEGSIAKMFDRVLEKEFSENDSPEGSDGAS   73
KEA6     7 RRRFSLSSQQLALLLLLLSFFLCFSVASPRAISDSDLLDETVANSSSSVASLNASSSSSLVKPKEGSFADIIDRALEKEFNESDQNEVAD   96
NhaS3    1                                                                 MFMNPLLPPLWPMIATAVETETEIAP   26

KEA1   568                                            (568) KRIKKLLEMIPHQEVNEEEASLFDFLWLLLA   598
KEA2   548                                            (548) KRIKKLLEMFPQQEVNEEEASLLDVLWLLLA   578
KEA3    77                                 (77) DTSKRFYFQGRWSESSGRRVETYAGVDVASAVDVINDLGFDTLTFLMV   114
KEA4    89 EVPDPGSFNNSVADQQAVLETVARVKPKKNETKTKEEKSFFNLDNENGVEDTPRLIDRKDNVFIMSNPKSKYPVLQLDLRLISDLVVVIV   178
KEA5    74 FNSSVADQQAEIETVAKVTHEKGKRNDTQENNGTRPFQLQDVFSLENEDSDDMTLIDKKNNVFVMSNKKSKYPILQVDLRLISDLVVIIV   163
KEA6    97 PGSFNNSVAGQQAVLETVARVKSTKKNETKEEKRFQLHDVFNLNNDNRAEDTPTLIDRKDNVFIISNSKSKYPVLQLDLRLISDLVVVIV   186
KefC     1                                                                        MDSHTLIQALIYLGS   15
NhaS3    1               MFMNPLLPPLWPMIATAVETETEIAPLVLAGVLLSLVVIYFASKLGGEVCLRLNLPPVLGELVGGVLVGVSAL   73

           TM1      TM2                                                          TM3
KEA1   599 SVIFVPLFQKIPGGSPVLGYLAAGILIGPYGLS--IIRNVHGTRAIAEFGVVFLLFNIGLELSVERLSSMKKYVFGLGSAQVLVTAAVVG   686
KEA2   579 SVIFVPLFQKIPGGSPVLGYLAAGILIGPYGLS--IIRNVHGTKAIAEFGVVFLLFNIGLELSVERLSSMKKYVFGLGSAQVLVTAAVIG   666
KEA3   115 TVIIVP-AFRILKASPILGFFFAGVVLNQFGL----IRNLTDVKVLSEWGILFLLFEMGLELSLARLKALAKFAFGMGLTQVLLCTAAFT   199
KEA4   179 SATCGGIAFACAGQPVITGYLLAGSIIGPGGLS--FVSEMVQVETVAQFGVIFLLFALGLEFSAAKLRVVRAVAIPGGLLQIFLFMCLSG   266
KEA5   164 FAAIGGIVFSCLGQPVIVGYLLAGSIIGPGGLK--FISEMVQVETVAQFGVVFLLFALGLEFSMTKLKVVGPVAVLGGLLQIVLLMFLCG   251
KEA6   187 SATCGGIAFACAGQPVITGYLLAGSIIGPGGLN--FISEMVQVETVAQFGVVFLLFALGLEFSTAKLKVVRSVAVLGGLLQILLFMFLCG   274
KefC    16 AALIVPIAVRLGLGS-VLGYLIAGCIIGPWGLR--LVTDAESILHFAEIGVVLMLFIIGLELDPQRLWKLRAAVFGGGALQMVICGGLLG   102
NhaS3   74 KLLLFPEGGLAPEDSLVIQLLMGSADLSPEAAQSVFSAQSEVISVISELGVIILLFEIGLESNLKELIRVGPQAAIVAVVGVVTPFSLGT   163

                                                     TM4                      TM5
KEA1   687 LLAH--------------------YVAGQAGPAAIVIGNGLALSSTAVVLQVLQERGESTSRHGRASFSVLLFQDLAVVVLLILIPLISP   756
KEA2   667 LITH--------------------YVAGQAGPAAIVIGNGLALSSTAVVLQVLQERGESTSRHGRATFSVLLFQDLAVVVLLILIPLISP   736
KEA3   200 AFELPPNGAIGTKILEFLFHSRPDLVNIRSIDEAVVIGAALSLSSSAFVLQLLAEKGELPTRFGSATLGILLLQDIAVVPLLVILPVLE-   288
KEA4   267 ITAS---------------------LCGGKLTEGIFVGAFLSMSSTAVVLKFLMERNSISALHGQITVGTLILQDCAVGLLFALLPVLG-   334
KEA5   252 VTAL---------------------LCGARLSEGIFVGAFLSMSSTAVVVKFLVERNSTSSLHGQVTIGILIFQDCVVGLLFALLPVLG-   319
KEA6   275 ITVS---------------------LCGGKRSEGVFVGAFLSMSSTAVVLKFLMEKNSTNSLHGQVTIGILILQDCAVGLLFALLPVLE-   342
KefC   103 LFCM--------------------LLG-LRWQVAELIGMTLALSSTAIAMQAMNERNLMVTQMGRRAFAVLLFQDIAAIPLVAMIPLLAA   171
NhaS3  164 IGLMT--------------------IFGVAAIPAIFAGAALTATSIGITAKVLAEINRLSSNEGQIIIGAAVLDDILGIIVLAVVGSLVK   233
              Clade Ib specific insert 1                                   Cation binding site

                   TM6                                                    TM7         TM8
KEA1   757 NSSKGGIGFQAIAEALGLAAVKAAVAITAIIAGGRL-----------LLRPIYKQIAENRNAEIFSANTLLVILGTSLLTARAGLSMALG   835
KEA2   737 NSSKGGIGFQAIAEALGLAAIKAAVAITGIIAGGRLFYNALIFWYTQLLRPIYKQIAENRNAEIFSANTLLVILGTSLLTARAGLSMALG   826
KEA3   289 ---SQDIGGESIWPMLAKESAKALGGLGILSLGGKF-----------FLRRIFEVVAETRSSEAFVALCLLTVAGTSLVTQWLGFSDTLG   364
KEA4   335 ----GTSGVLQGVLSMAKSLAILIAFLGALFVLSRT---------WVPWFLKLMTSLSSQTNELYQLAAVAFCLLVAWCSDKLGLSLELG   411
KEA5   320 ----GNSGLLQGIISMGKLLLILSIYLTVASLLTWS---------FVPRFLKLMIQLSSQTNELYQLAAVAFCLLSAWCSDKLGLSLELG   396
KEA6   343 ----GNSGIVHGMLSIGKVVVLLLSFLAVLSILSRT---------CIPWLLKLMVSLSSQTNELYQLAAVAFCLLVAWCSDKLGLSLELG   419
KefC   172 SSASTTMG------AFVLSALKVAGALVLVVLLGRY-----------VTRPALRFVARSGLREVFSAVALFLVFGFGLLLEEVGLSMAMG   244
NhaS3  234 -------TGEIQISNIIYLILSATGFVVGSILIGRL-----------LSPFYVSLVNRMKTRGQLLLVSICVAFVLSYIAQIVQLEAILG   305

                       TM9                                         TM10
KEA1   836 AFLAGLLLAETEFSLQVESDIAPYRGLLLGLFFMTVGMSIDPKLLLSN-------FPVIVGTLGLLIVGKTMLVVIMGKLFGISIISAIR   918
KEA2   827 AFLAGLLLAETEFSLQVESDIAPYRGLLLGLFFMTVGMSIDPKLLLAN-------FPLIMGTLGLLLVGKTILVVIIGKLFGISIISAVR   909
KEA3   365 AFLAGALLAETNFRTQIEADIRPFRGLLLGLFFVTTGTSIDMEVLFRE-------WPNVLSLLGGLIVIKTLIITAIGPRVGLTIQESVR   447
KEA4   412 SFAAGVMISTTDLAQHTLEQVEPIRNFFAALFLASIGMLIHMHFLWNH-------VDILLAAVLLVIVIKTVVVAIVVKVFGYNNKTAVL   494
KEA5   397 SFVAGVMLSTTEFAQHTLEQVEPIRNLFAALFLSSIGMLINVHFLWNH-------VDILLASVILVIVIKTAIAAVVVKAFRYNMRISFH   479
KEA6   420 SFAAGVMISTTDLAEHTLEQIEPIRNLFAALFLASIGMLVNVHFLWTH-------VDILLASVILVIIIKTTIVTTVVKGFGYNNKTALL   502
KefC   245 AFLAGVLLASSEYRHALESDIEPFKGLLLGLFFIGVGMSIDFGTLLENP-------LRIVILLLGFLIIKIAMLWLIARPLQVPNKQRRW   327
NhaS3  306 SFAAGLILAETEKREDLEEQILPLADFFVPVFFVCVGAKTDVSVLNPAVPANREGLIIAAFLILVAIVGKVVTGFTLFGKSELNKLAIGV   395
                           regulatory loop                           dpgr point mutation site

                   TM11                                                                      *
KEA1   919 VGLLLAPGGEFAFVAFGEAVNQGIMSPQLSSLLFLVVGISMAITPWLAAGGQL----------------IASRFELHDVRSLLPVESETD   992
KEA2   910 VGLLLAPGGEFAFVAFGEAVNQGIMTPQLSSLLFLVVGISMALTPWLAAGGQL----------------IASRFELQDVRSLLPVESETD   983
KEA3   448 VGFLLSQGGEFAFVVFSLANRLGVLPNELNKLLIIVVVLSMALTPYLNQLG------------------LTPYLNQLGRKAADFLDERLD   510
KEA4   501 VGMSLAQIGEFAFVLLSRASNLHLIESKLYLLLLGTTALSLVTTPLLFKLIPAVVHL--------------------------------   551
KEA5   488 VGVLLAQIGEFAFVLLSRASNLHVIEGKMYLLLLGTTALSLVTTPLLFKLIPSAMNL--------------------------------   536
KEA6   509 VGISLAQIGEFAFVLLSRASNLHLIEGKLYLLLLGTTALSLVTTPLVFKMIPAVVHL--------------------------------   559
KefC   334 FAVLLGQGSEFAFVVFGAAQMANVLEPEWAKSLTLAVALSMAATPILLVILNRL----------------EQSSTEEAREADEIDEEQP   417
NhaS3  396 ---GMIPRGEVGLVFAGVGAASGALDPATDAAIIVMVIVTTFVAPPWLRAVFEGAKKEEAPEKPVPTPD---------------------   461

                                                 KTN domain
KEA1   993 -----------DLQGHIIICGFGRVGQIIAQLLSERLI--------PFVALDVSSDRVTIGRSLDLPVYFGDAGSKEVLHKIGAGRACAA   1063
KEA2   984 -----------DLQGHIIICGFGRIGQIIAQLLSERLI--------PFVALDVSSDRVAIGRSLDLPVYFGDAGSREVLHKIGADRACAA   1054
KEA3   511 PGEKIGEDVNFDVSESIVIIGFGQMGQVLANFLSTPLVSDSDLVGWPYIGFDLNPAVVKESRKLGFPILYGDGSRPSVLQSAGVSSPKAI   600
KefC   418 ---------------RVIIAGFGRFGQITGRLLLSSGV--------KMVVLDHDPDHIETLRKFGMKVFYGDATRMDLLESAGAAKAEVL   467
                        Rossman fold   Clade Ib specific insert 2

                     KTN domain
KEA1  1064 VVALDAPGANYRCVWALSKFYPNVKTFVRAHDVVHGLNLEKAGATAVVPETLEPSLQLAAAVLAQAKLPTSEIANTINEFRTRHLSELTE   1153
KEA2  1055 AIALDTPGANYRCVWALSKYFPNVKTFVRAHDVDHGLNLEKAGATAVVPETLEPSLQLAAAVLAQAKLPTSEIATTINEFRSRHLSELAE   1144
KEA3   601 MIMYKGKKRTTEAVQRLRLAFPGSPIYARAQDLPHLLELKKAGATDAILENAETSLQLGSKLLTGFGVMSDDVSFLSKVFRDSMEIQAQE   690
KefC   468 INAIDDPQTNLQLTEMVKEHFPHLQIIARARDVDHYIRLRQAGVEKPERETFEGALKTGRLALESLGLGPYEARERADVFRRFNIQMVEE   557


KEA1  1154 LCEASGSSLGYG-----------------YSR-TSKPKP-QPSDASGD-------------NQIIEGGTVVI-----------------   1193
KEA2  1145 LCEASGSSLGYG-----------------FSRSTSKPKPPSPSETSDD-------------NQIIEG-TLAI-----------------   1185
KEA3   691 EITASETNAGLKPMQMKASDINVVSAATQKQVQLMKPMQMKASDSNSDSAAEILQETAGLSQPPEIDDSSVNIDNGFVGKADKAQD----   777
KefC   558 MAMVENDTKARA---------------AVYKRTSAMLSEIITEDREHLS----------LIQRHGWQGTEEGKHTGNMADEPETKPSS--   620
```

Supplementary figure S1. Multiple sequence alignment of the amino acid sequences of Arabidopsis KEA1-KEA6, *E. coli* KefC and *Synechocystis* NhaS3 showing predicted transmembrane domains and the KTN domain.

Predicted TMs were assigned according to the X-ray crystal structures of *E. coli* NhaA[33] and *Thermus thermophilus* NapA[34]. Multiple amino acid sequence alignment was generated by ClustalW. The number of N-terminal amino acids removed from KEA1-KEA3 is shown in parentheses. The conserved aspartate-aspartate and glutamate-aspartate motifs are shown in red. The conserved lysin residue (bold, in blue) near G422 in KEA3, the mutated residue in the Arabidopsis *dpgr* mutant (bold, in green) are marked. The asterisk indicates the residue at position 509 in KEA3.

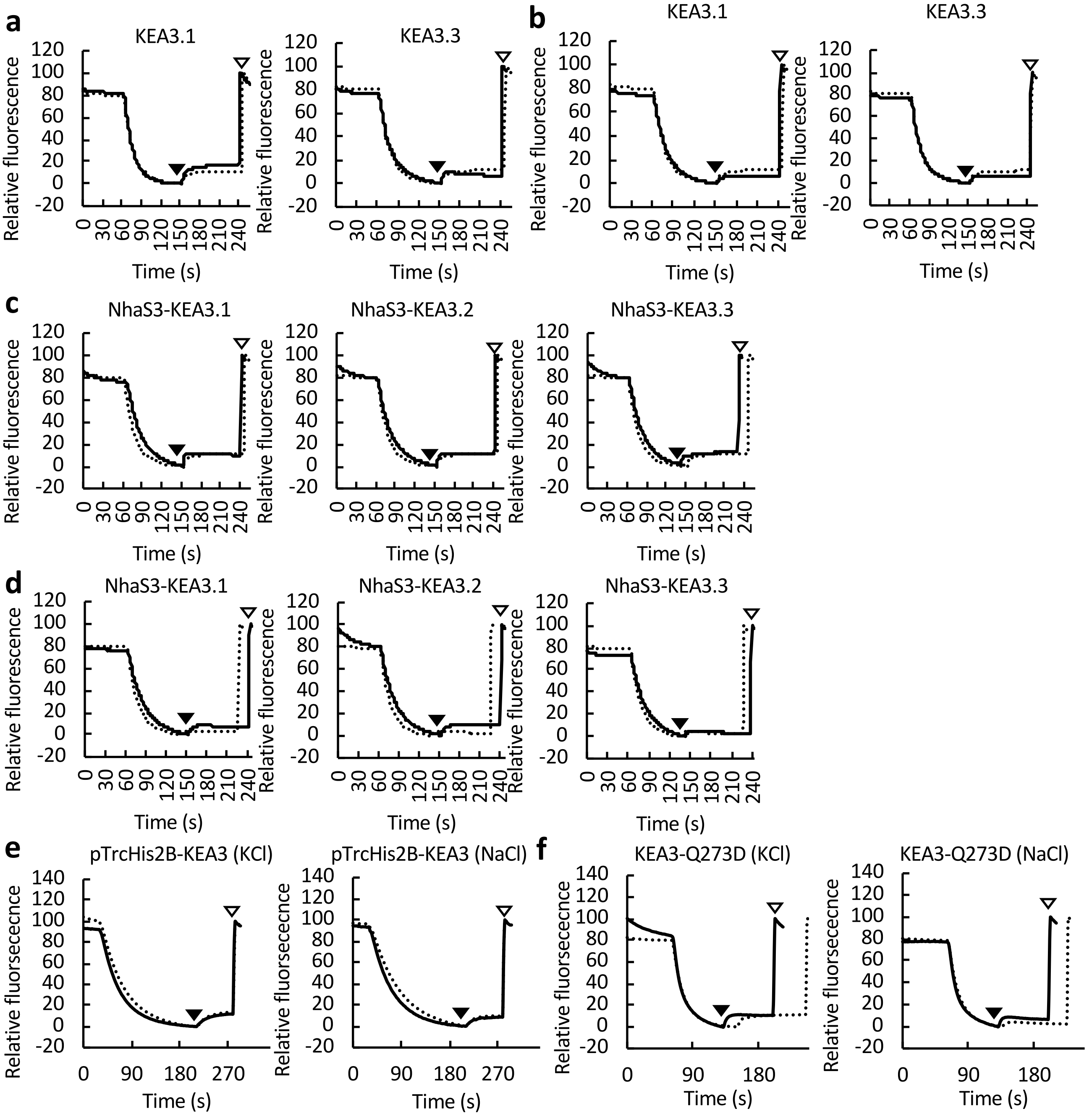

a
KEA3.1
KEA3.3
b
KEA3.1
KEA3.3
c
NhaS3-KEA3.1
NhaS3-KEA3.2
NhaS3-KEA3.3
d
NhaS3-KEA3.1
NhaS3-KEA3.2
NhaS3-KEA3.3
e
pTrcHis2B-KEA3 (KCl)
pTrcHis2B-KEA3 (NaCl)
f
KEA3-Q273D (KCl)
KEA3-Q273D (NaCl)
Relative fluorescence
Relative fluorsececnce
Time (s)

Supplementary figure S2. K^+/H^+ and Na^+/H^+ antiporter activity of KEA3, KEA3 splice variants and the corresponding chimeric proteins NhaS3-KEA3

(a), (b) K^+/H^+ antiporter activity (a) and Na^+/H^+ antiporter activity (b) of KEA3.1, KEA3.3 were measured in everted vesicles prepared from TO114 transformants. The data of *E. coli* containing pPAB404 are the same as in Fig. 3a and b.

(c), (d) K^+/H^+ antiporter activity (c) and Na^+/H^+ antiporter activity (d) of chimeric proteins of NhaS3-KEA3 (NhaS3-KEA3.1, NhaS3-KEA3.2 and NhaS3-KEA3.3) was measured in everted vesicles prepared from TO114 transformants. The data for *E. coli* containing pPAB404 (broken lines) are the same as in Fig. 3a and b.

(e) K^+/H^+ antiporter activity and Na^+/H^+ antiporter activity measured in vesicles derived from TO114 containing pTrcHis2B-KEA3.2. The data for *E. coli* containing pPAB404 (broken lines) are the same as in Fig. 3a and b.

(f) K^+/H^+ antiporter activity and Na^+/H^+ antiporter activity was measured in vesicles derived from TO114 strain containing pPAB404-KEA-Q273D. The data for *E. coli* containing pPAB404 (broken lines) are the same as in Fig. 3a and b.

The symbols and lines are same as in Fig. 3.

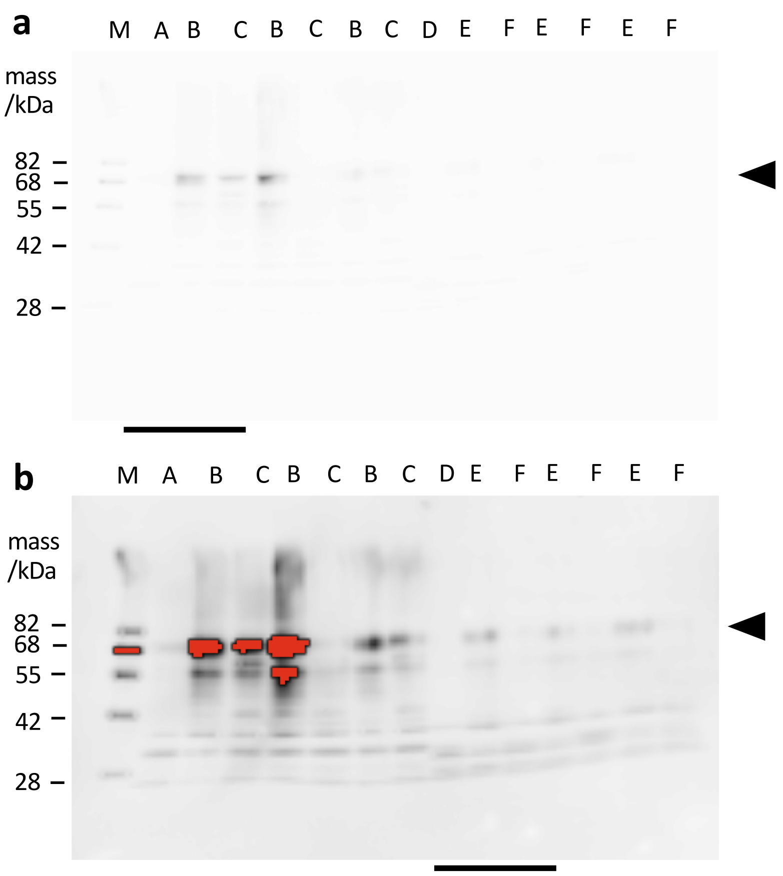

Supplementary figure S3. Full images of immunoblot detection of KEA3-His-tag and KEA3-G422R-His-tag proteins.

Short time exposure (a) and long time exposure (b) images of immunoblot detection were used for Fig. 7b and Fig. 7c, respectively. Dr. Western (ORIENTAL YEAST CO.,LTD.) (M), membrane fractions from TO114 containing pTrcHis2B (A), pTrcHis2B-KEA3-His (B), pTrcHis2B-KEA3-G422R-His (C), LB2003 containing pTrcHis2B (D), pTrcHis2B-KEA3-His (E) and pTrcHis2-KEA3-G422R-His (F) were loaded onto the gel. Samples from the triplicated experiments were loaded. The position of KEA3/KEA3G-422R band is indicated by the arrow. Bar under the blots indicate the position of the data represented in Figure 7.
